# Supplementary material for: An association between prediction errors and risk-seeking: Theory and behavioral evidence
Source: PLoS Comput Biol. 2021 Jul 16;17(7):e1009213. doi: 10.1371/journal.pcbi.1009213 (PMC8318232; doi:10.1371/journal.pcbi.1009213)
Supplement: S1 Text — (DOCX) [file pcbi.1009213.s001.docx]

# Supporting information

Pupillometry

In addition to choices, we also recorded our participant’s pupil diameter during the task. Here, we use the well documented link between pupil dilation and surprise to consolidate our theory (Preuschoff, ’t Hart et al. 2011, Cavanagh, Wiecki et al. 2014, Browning, Behrens et al. 2015, Lawson, Mathys et al. 2017). If participants experience stimulus prediction errors at stimulus onset and outcome prediction errors at reward onset, (i.e., deviations from their expectations), it is likely that they should also experience surprise at those times, proportionally to the magnitude of the prediction error. Our theory thus implies that pupils might respond to the magnitude of the two prediction errors.

We tested this, using linear regression methods. We find pupil responses to the magnitude of both the stimulus and the outcome prediction error, indicating that those indeed registered as surprising events. While these results do not prove the mechanistic part of our explanation centered around dopamine, they still provide support for the cognitive mechanisms we hypothesized.

Pupil dilation reflects outcome prediction errors

To test whether pupils responded to outcome prediction error magnitude, we extracted trial-by-trial estimates of the outcome prediction error $\left| \delta_{outcome} \right|$from the PEIRS model fits. Regression analyses were used to determine whether pupil dilation after reward presentation encoded$|\delta_{outcome}|$. We found a phasic response which peaked 0.9 s after reward presentation (Fig A, panel A). This suggests that surprise about the outcome was indeed represented in pupil dilation traces.


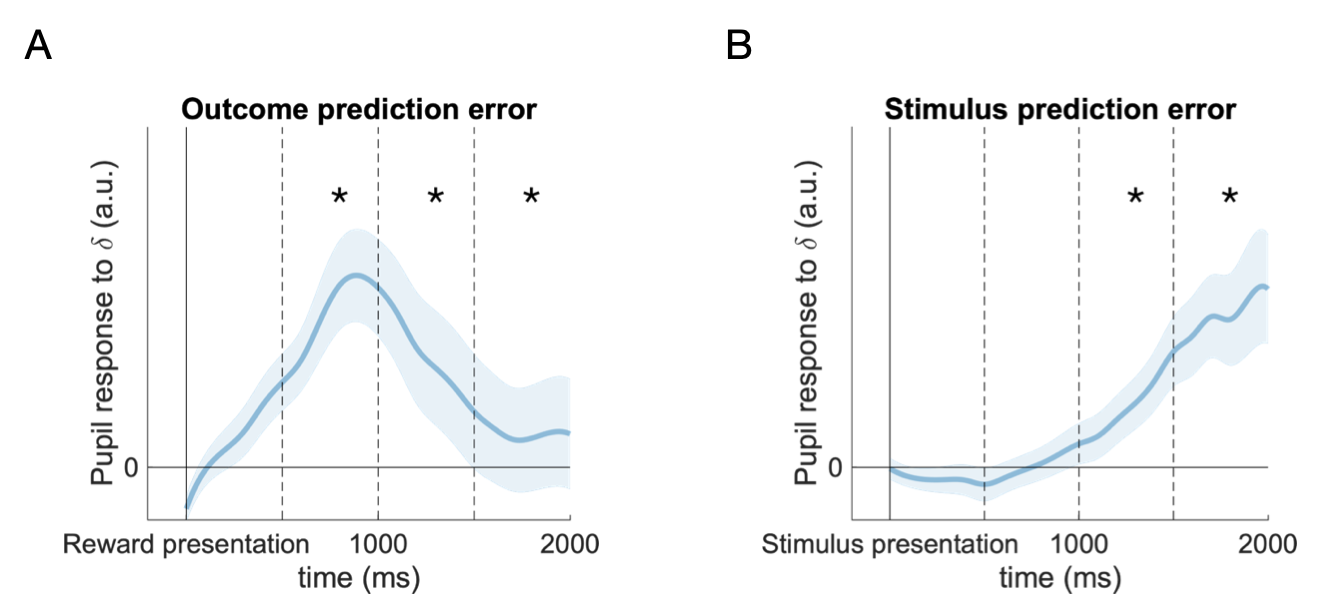


Fig A: Pupil responses to prediction error magnitude

A, B) Pupil dilation encodes the magnitude of the outcome prediction error and the stimulus prediction error. The blue lines and shadings represent the population means and SEs of the pupil response to prediction error magnitude, estimated with a mixed-effects linear model. Pupil dilation was predicted from trial-by-trial estimates of prediction errors, obtained from a fitted PEIRS model. Responses were aligned at reward presentation (A) or stimulus presentation (B). For display, the traces were smoothed using spline interpolation. Asterisks mark 500 ms time bins in which the pupil responses were significantly larger than zero (one-tailed t-tests, Bonferroni corrected, $\alpha=0.05$).

Pupil dilation reflects stimulus prediction errors

Next, we tested whether pupils reflect stimulus prediction error magnitude. For this, we extracted estimates of $\left| \delta_{Stimulus} \right|$ for every trial and used an analysis similar to the previous step to extract the corresponding pupil response. Here, we aligned the pupil signal at stimulus presentation, and censored all data points collected after reward presentation to avoid confounding factors such as reward or outcome prediction errors. We found a phasic response staring about 1 s after stimulus onset (Fig A, panel B). This suggests that surprise related to the stimulus prediction error is represented in the pupil during the period of choice, which is consistent with the existence of a stimulus prediction error that occurs after stimulus onset and might modulate preferences.

The onset of the response to the stimulus prediction came later than that of the outcome prediction error. There might be many reasons for this difference in delay. Among those, differences in information processing might play a role: generating a stimulus prediction error involves two stimuli, hence attention mechanisms, in addition to retrieval of value estimates from memory. Generating the outcome prediction error, on the other hand, just requires the processing of a number.

Pupillometry discussion

We found pupil responses to the magnitude of both the stimulus prediction error and the outcome prediction error. Those might reflect the surprise associated with the two prediction errors that we have postulated and might hence constitute an indirect physiological correlate of the mechanisms we have described. Note that we do not claim that pupils should be thought of as a proxy for dopamine, in terms of a direct physiological link—in fact, pupils are perhaps more likely to reflect noradrenalin, see e.g. (Reimer, McGinley et al. 2016).

Noradrenalin has been linked to uncertainty in an influential model (Yu and Dayan 2005). In that model, two types of uncertainty are identified: expected uncertainty (known unreliability of cues) and unexpected uncertainty (surprises following previously reliable cues). Expected uncertainty is associated with acetylcholine, which is discussed in the context of cortical and hippocampus learning. Unexpected uncertainty is linked to noradrenalin. The uncertainty in our task is of the expected variety—our theory even suggests that participants learn how reliable cues are with respect to the reward they predict. Unexpected uncertainty could be introduced into our task through reversals or other sudden changes but does not feature in the current design. We still detect pupil responses to (expectedly) surprising outcomes. Since pupils are linked to noradrenalin, it appears that our results are slightly at odds with the model of (Yu and Dayan 2005). Further work is required to dissect the different kinds of uncertainty, risk and surprise, the role of the various neurotransmitters and the relation to pupil dilation.

For the intends and purposes of this study, we propose that pupil responses might reveal cognitive states such as surprise, which in turn are related to DA release according to the widely accepted reward prediction error hypothesis. The link between pupil traces and DA is thus weak, which is why this analysis is a supplement to our main results, rather than a main result itself. Nevertheless, the pupil results together with our other findings yield a consistent picture.

Pupillometry methods

We recorded time series of pupil diameters for every trial, using an EyeLink 1000 system. For each participant, the system was calibrated before the first block, and after subsequent blocks if required.

The raw measurements were screened for blinks, which were identified as missing values in the recordings. Data points in the direct vicinity of blinks as well as data segments shorter than 50 ms were removed. After cleaning, missing value segments of less than 400 ms were filled using linear interpolation (Manohar 2019). Then, the traces were aligned to the relevant temporal markers (stimulus onset, or reward onset). We used the mean over 500 ms prior to the alignment point to define a trial-wise baseline. All traces were divided and shifted by that baseline, resulting in traces reflecting the relative change of pupil diameter after the alignment point. Finally, traces were downsampled to 10 Hz.

Pupil responses to prediction errors were obtained using mixed effect linear models: we regressed pupil dilation against a trial-by-trial estimate of the prediction error magnitude of interest, which was obtained from a model fit. We accounted for individual differences by including random effects for the intercept and the prediction error regressor. We further included the stimulus identity—i.e., the fractal picture that was used—of the chosen and the unchosen stimulus as control regressors. Regressions were run for each time bin of the pupil signal and resulted in a pupil response time course (beta weights and standard errors as functions of time) for the population effect.

To uncover the pupil response to the stimulus prediction error, we aligned the pupil time courses at stimulus onset. After stimulus onset, participants would eventually make a choice (with variable delay, the median reaction time was 0.86 s) and receive a reward (with a 1 s delay) after their choice. Since the reward or the resulting outcome prediction error might confound our regression analysis, we censored out all data after reward presentation. This means that the number of observations on which regressions can be based rapidly declines after the median reward presentation time, which is at 1.86 s after stimulus onset. Estimates obtained later are increasingly unreliable, since they are based on insufficient data. We hence conducted our analyses for the interval 0 s to 2 s after stimulus onset. This allows us to obtain reliable estimates of the statistics, while still avoiding confounding effects related to reward presentation.

To test whether the pupil responses to the prediction errors are statistically significant, we followed (Browning, Behrens et al. 2015): we first split the pupil traces in four sequential 500 ms time bins. We then conducted a one-tailed t-test in each of these bins and applied the Bonferroni correction for multiple comparison to the results of the individual tests.

Performance

To be included in our analysis, participants had to choose the high value option over the low value option at least in 65 % of the relevant trials across the experiment. This threshold was chosen to separate two clusters of participants that occurred in our cohort—learners and non-learners (as can be seen in Fig B, there are 3 participants that perform substantially worse than the other 27).


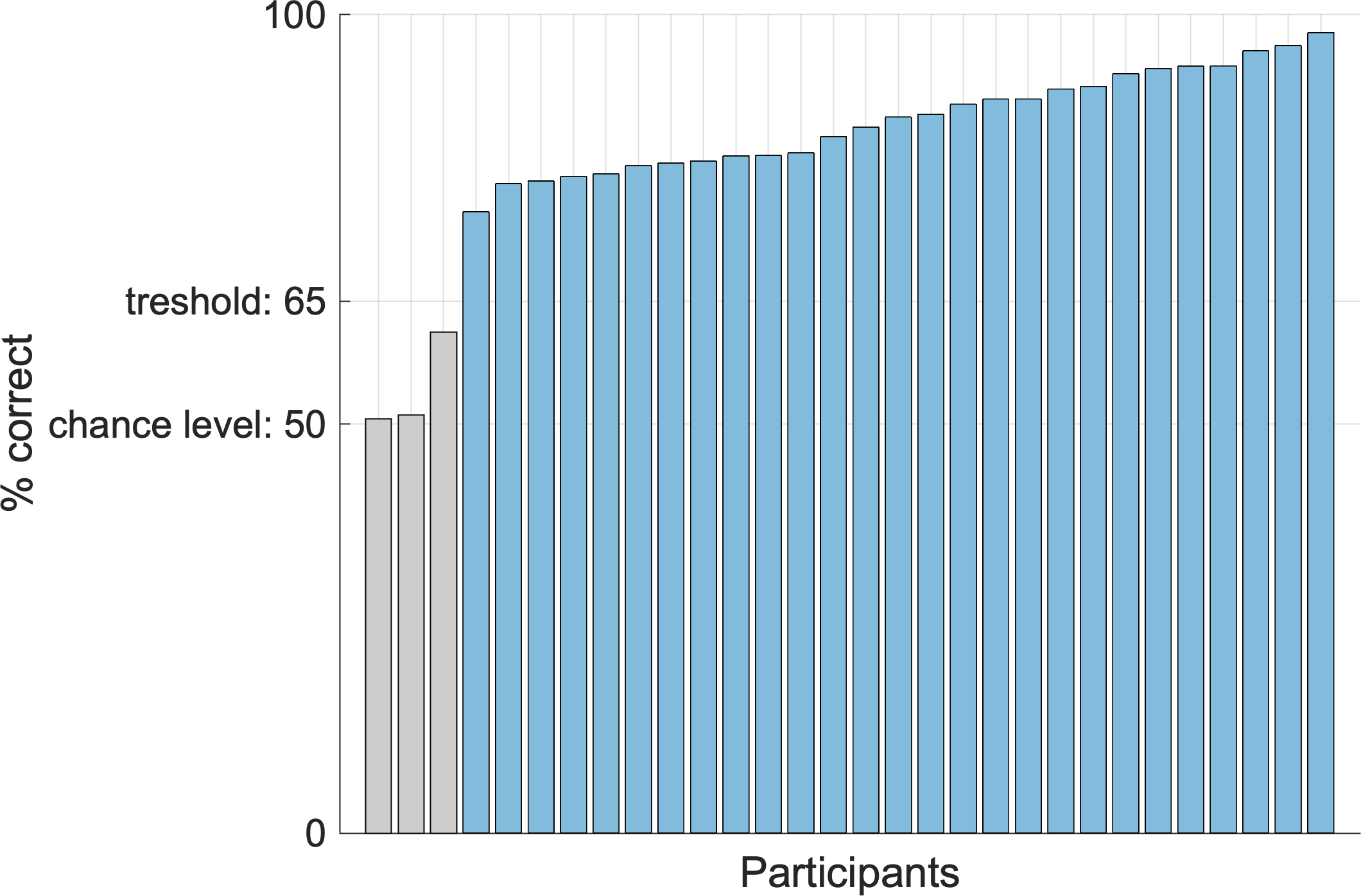


Fig B: Performance

We evaluated participant’s mean performance (the relative frequency of choosing a high-mean stimulus over a low-mean stimulus in the “different” condition) across all four blocks. Participants with performances below 65 % were excluded from our analyses (grey bars), on the grounds that they had not understood or engaged in the task. Participants with performances over 65 % were included in our analyses (blue bars).

Choice probabilities in the different condition

To complement the results shown in Fig 2, we provide the choice probabilities in the difference condition. The difference condition encompasses four different types of choices: either low-risky or low-safe is paired with either high-risky or high-safe. We show results for all those combinations.


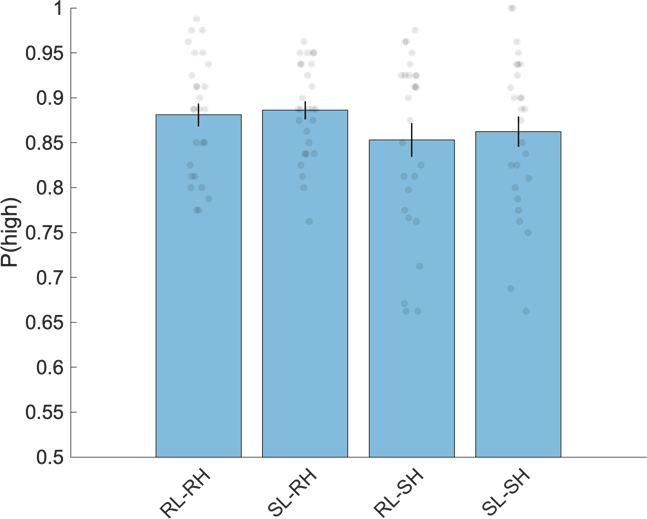


Fig C: Choice probabilities in the different condition. We show the average probability of choosing the high-valued option, for all combinations of stimuli (RL: risky-low, SL: safe-low, RH: risky-high, SH: safe-high). The error bars indicate the SE of the mean across participants (N=27), the grey dots show individual participants.

Model likelihoods of risk preferences


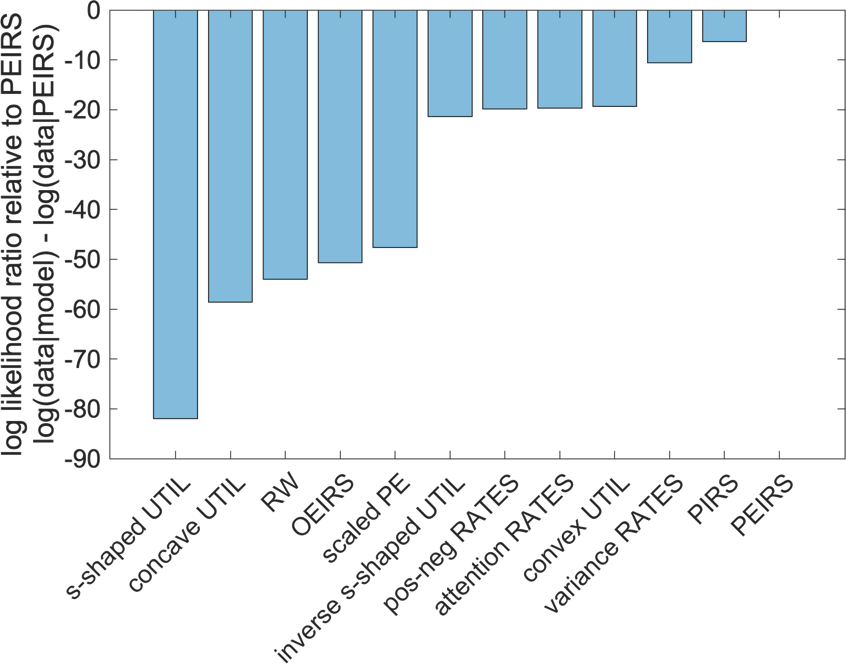


Fig D: Model likelihoods of risk preferences

To quantify how well the simulated distributions (blue shadings in Fig 3A) match the empirical distribution (grey dots in Fig 3A), we used MATLAB’s kernel density estimation (ksdensity) to interpolate the simulated distributions and hence obtain likelihoods for the empirical risk preferences. In other words, we used the simulated distributions to estimate how likely the empirical risk preferences were given each model. We then computed the log likelihood ratios relative to PEIRS

$$LLR_{all rp}\left( model, PEIRS \right)=\sum_{participant i} \log\left( rp_{i} | model \right)-log(rp_{i}|PEIRS)$$

Eq. S1

for all models (blue bars). The diagram shows that the empirical risk preferences are most likely under PEIRS, and least likely under s-shaped UTIL.

Additional Simulations

Above, in Fig 3A, B and C and in Results/Modelling/Simulations, we present results based on simulations of a group of models that we considered the most relevant alternative explanations. We performed the same analysis for all other candidate models as well—they are presented in Fig E.


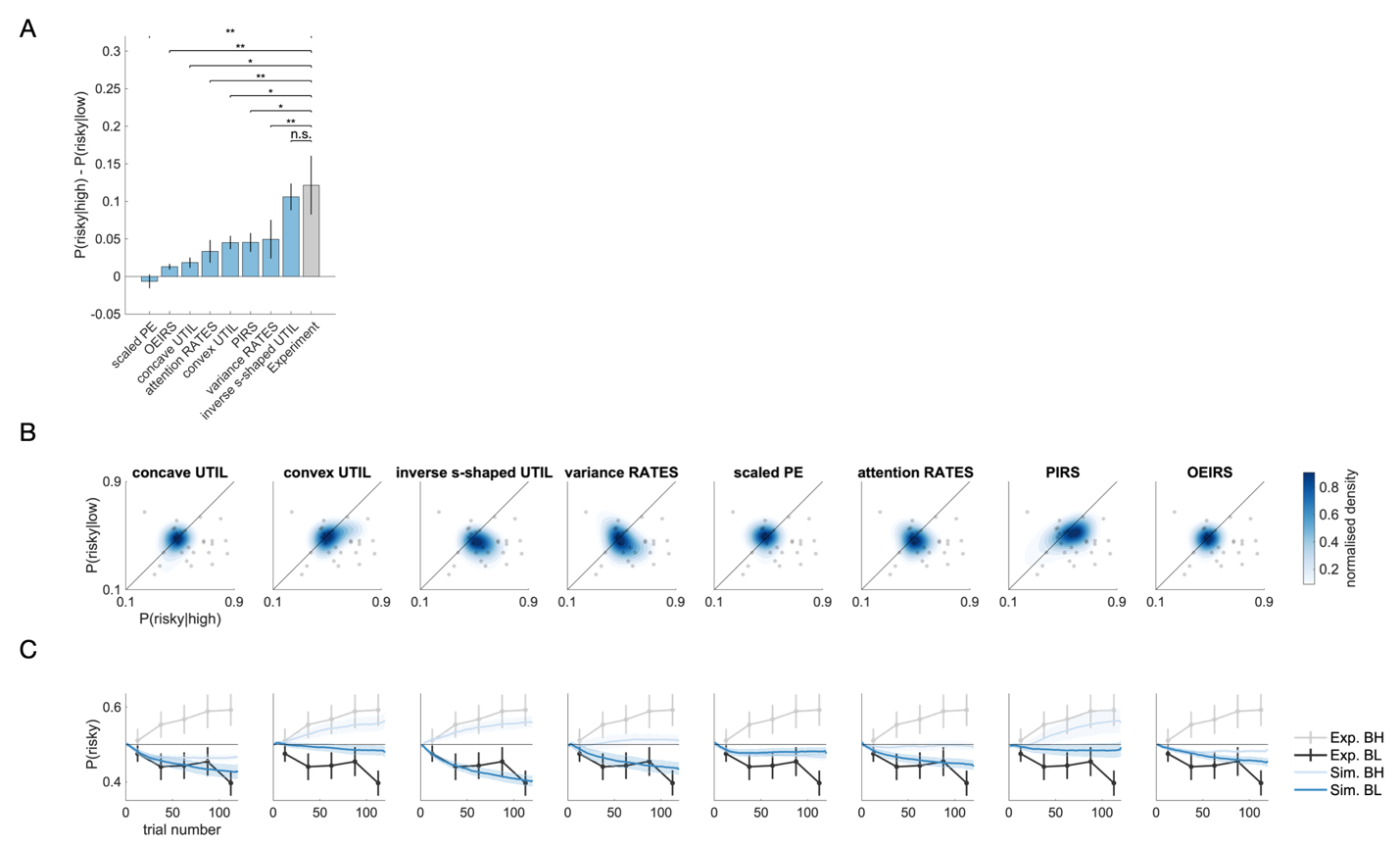


Fig E: Additional simulations. The representation of data in panels A, B and C is completely analogous to the presentation in panels C, A and B of Fig 3. We performed the same analysis as in Fig 3, but on a different set of models: concave UTIL, convex UTIL, inverse s-shaped UTIL, variance RATES, scaled PE, attention RATES, PIRS and OEIRS.

Model recovery

To validate our model selection procedures, we performed a model recovery analysis (Palminteri, Wyart et al. 2017). This analysis is meant to show whether our candidate models are identifiable in the empirical parameter range. We hence performed an a posteriori recovery analyses: first, models were fitted to the empirical dataset, which resulted in posteriors over the parameter space. Parameters were then sampled from those posterior distributions, and simulations were performed with the sampled parameters. Finally, all models were fitted to all simulated datasets, and model selection metrics (BIC) were extracted. This results in a confusion matrix. We repeated this procedure 100 times for each of our 27 participants (a total of 2700 repetitions) to account for stochastic fluctuations. The averaged confusion matrix is shown in Fig F.


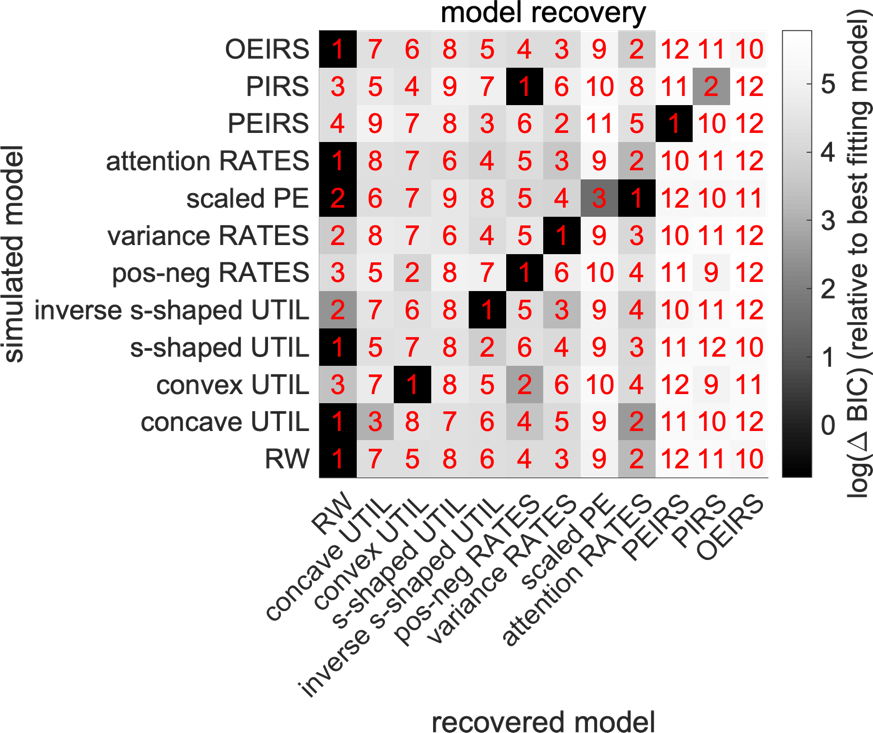


*Fig F: Model recovery confusion matrix. Each row corresponds to a model used to simulate datasets. Each column corresponds to a model fitted to various datasets. The shading of the matrix elements corresponds to the BIC relative to the best fitting model (BICs were baselined per row). Higher BICs correspond to lighter colors and to poorer fits. The color scale is logarithmic to enhance contrasts. The red numbers indicate the rank of the model in the model comparison per row (1 meaning best fitting model, 12 meaning worst fitting model).*

The confusion matrix suggests that 6 out of 12 models (RW, convex UTIL, inverse s-shaped UTIL, pos-neg RATES, variance RATES and PEIRS) can be directly identified by our procedure, with ground truth simulations based on the empirical parameter range. For another 5 out of 12 models (Concave UTIL, s-shaped UTIL, scaled PE, attention RATES, OEIRS), RW was selected as the better model to describe the datasets they generated. Given that all those models contain RW as a special case, this means that the additional effects they describe just do not occur in our dataset—their empirical parameter range is such that the models mimic RW and make no use of the additional features they can describe. The model comparison still penalizes the additional complexity, leading to RW as the selected model. The only model that is confused with a model other than RW is PIRS, which is confused with pos-neg RATES. This means that for all models but PIRS, our model comparison will either select the correct model if an effect is there or the base model (RW) if the effect does not appear. Importantly, we find that in the empirical parameter range, PEIRS is selected unambiguously. This suggests that PEIRS being selected in the model comparison on the empirical dataset is a meaningful result.

# Reference

Browning, M., et al. (2015). "Anxious individuals have difficulty learning the causal statistics of aversive environments." Nature neuroscience **18**(4): 590-596.

Cavanagh, J. F., et al. (2014). "Eye tracking and pupillometry are indicators of dissociable latent decision processes." Journal of Experimental Psychology: General **143**(4): 1476.

Lawson, R. P., et al. (2017). "Adults with autism overestimate the volatility of the sensory environment." Nature neuroscience **20**(9): 1293.

Manohar, S. (2019). Matlib: MATLAB tools for plotting, data analysis, eye tracking and experiment design (Public).

Palminteri, S., et al. (2017). "The importance of falsification in computational cognitive modeling." Trends in cognitive sciences **21**(6): 425-433.

Preuschoff, K., et al. (2011). "Pupil dilation signals surprise: Evidence for noradrenaline’s role in decision making." Frontiers in neuroscience **5**: 115.

Reimer, J., et al. (2016). "Pupil fluctuations track rapid changes in adrenergic and cholinergic activity in cortex." Nature communications **7**(1): 1-7.

Yu, A. J. and P. Dayan (2005). "Uncertainty, neuromodulation, and attention." Neuron **46**(4): 681-692.
