# Supplementary material for: An association between prediction errors and risk-seeking: Theory and behavioral evidence
Source: PLoS Comput Biol. 2021 Jul 16;17(7):e1009213. doi: 10.1371/journal.pcbi.1009213 (PMC8318232; doi:10.1371/journal.pcbi.1009213)
Supplement: Attachment — Submitted filename: Response to reviewers.pdf [file pcbi.1009213.s002.pdf]

Response to the reviewers

For the manuscript "An Association Between Prediction Errors and Risk-Seeking: Theory and Behavioral Evidence".

Moritz Moeller, Jan Grohn, Sanjay Manohar & Rafal Bogacz.

We are grateful for the comments from all three reviewers. Their insightful suggestions helped us improve our manuscript considerably and we are happy to present our updated results here. Below we are responding to each comment in turn. The reviewers' original points are shown in italics while our responses are shown in blue ink. Sections of the manuscript and supporting information are shown in a standard font with new areas highlighted.

Reviewer #1

Moeller et al attempt to unify two classic functions attributed to dopamine neurons – reward prediction errors and risk preferences. Building off of beautiful and rigorous computational work by the same authors (Mikhael and Bogacz 2016; Moller and Bogacz 2019), the authors develop a neurally-inspired theory that attempts to unify these two functions. Motivated by the theory, they develop a two-alternative forced choice task and find behavioral and pupillometric evidence in support of their theory.

Overall, I find the task design and behavioural analysis interesting, compelling, and non-trivial. However, I remain rather unconvinced by the theory, the computational modelling, and the pupillometric data, and I believe a substantial amount of work would be needed to make them compelling.

> Thank you for reading our manuscript so thoroughly and for the points you raised. We agree with a lot of the issues you highlight and hope that our comments address them appropriately.

Major Comments

1 I find the extension of the theory in this manuscript a bit arbitrary. The theory is developed from the perspective of basal ganglia circuitry and the authors posit the existence of action channels (Ai) modulated by direct pathway excitation (Gi) and indirect pathway inhibition (Ni). The activation of these channels is proportional to the probability of choosing option i. The authors then develop Eq. 1 as a consequence of dopamine levels of above/below baseline. However, one can rewrite Eq. 1 as follows:

$Ai=(\delta+0.5)Gi + (\delta-0.5)Ni$

Written this way, if the system receives $\delta>0.5$, then Ni, the indirect pathway inhibition, now increases the probability of choosing Ai. This appears counterintuitive to me, given the description of the system. It seems to me that the equation written in line 177, $Ai=Qi + \delta Si$, is a more natural starting point as a hypothesis for how the system may function, with Eq. 1 as a consequence. However, if this is a starting point, I do not see a priori why this particular functional form was chosen and not any other (for example, $Ai=Qi - \delta Si$, to allow for risk

aversion for gains and risk preference for losses). In other words, there seems to be enough flexibility with the development of the theory to allow it to fit the empirical data at hand. Unless I am missing something, this flexibility greatly limits the ability to interpret the behavioural results as a natural consequence of the basal-ganglia-inspired theory.

The reviewer points out an important issue that we failed to address in our exposition: if the dopamine deviation from baseline δ in Eq. 1 of the original submission takes on values outside of -0.5 and 0.5, paradoxical situations arise (the reviewer points to a situation in which inhibition from the indirect pathway makes an action more likely). The reviewer suggests choosing another starting point for the theory but also raises the concern that that starting point may be somewhat arbitrary, limiting the explanatory power of the theory.

We strongly agree that there is an unresolved issue, and propose a simple solution: First, we propose to take the equation

$$A_i = DG_i - (1-D)N_i$$

as our starting point, where D is the dopamine concentration, which takes values between 0 and 1. This equation is developed in [1] (see equation 25), and is designed to capture the main dependencies of the BG output. The biological foundations of the equation are discussed in detail in the reference.

Second, to make sure that we stay within the biologically plausible regime (i.e. N_i is always inhibiting and G_i is always exciting), and to avoid any paradoxical situations, we change our models such that the variable D stays within the required range of [0,1]. This is achieved by writing

$$D = \sigma(\omega\delta)$$

where δ can take any value, and σ is a sigmoid function. This effectively models a saturation effect--the modulation of the pathway balance converges to its maximal state as dopamine levels rise.

We changed the manuscript such that this modification is introduced in our theory section and in the relevant model descriptions, and implement it in all models based on Eq. 1 (i.e., PEIRS and all variants thereof).

While this change of the models causes small quantitative changes in the fits and the derived metrics (i.e. slightly different p-values etc), it does not change any major result.

Specifically, in the theory section of the manuscript we now write

> These contributions are differentially modulated by dopamine (DA), mediated through the D1 and D2 receptors expressed in the direct and indirect pathway respectively. ~~Let δ represent the deviation of DA levels from baseline (i.e., $\delta = 0$ means baseline DA levels, $\delta >$ 0 means DA is above baseline etc.)~~. Let $D \in [0,1]$ represent the level of dopaminergic modulation ($D = 0.5$ is the baseline level, which corresponds to a balance between direct and indirect pathway. $D = 1$ means that modulation is such that only the direct pathway contributes to action selection, while $D = 0$ means that only the indirect pathway contributes.). Then, the activation of an action channel corresponding to option i can be approximated as
>
> $$A_i = D \times G_i - (1 - D) \times N_i.$$
>
> Eq. 1
>
> This formula is motivated by (Möller and Bogacz 2019), Eq. 25, where a detailed discussion of biological plausibility is provided.

In the same section, we then go on to explain that

> As mentioned above, D must take values between 0 and 1 for Eq. 1 to be biologically plausible. To ensure this, we introduce a reparameterization: let $\delta \in (-\infty, +\infty)$ quantify dopamine release relative to the baseline, i.e., $\delta = 0$ corresponds to steady

state dopamine release, $\delta < 0$ means that dopamine release is suppressed, and $\delta > 0$ means that dopamine release is enhanced. We can then write

$$D = \sigma(\tilde{\omega}\delta)$$

Eq. 5

with $\sigma(x) = (1 + e^{-x})^{-1}$ the sigmoid function and $\tilde{\omega}$ a proportionality constant. Using this notation, we can be sure that Eq. 1 will produce biologically plausible results for any value δ may assume. Inserting the new parametrization into Eq. 4 yields

$$A_i = Q_i + tanh(\omega\delta) \times S_i,$$

Eq. 6

with $\omega = \tilde{\omega}/2$ a rescaled proportionality constant. This is the equation that we will use in our models. As we have shown, it is derived from a model of the basal ganglia pathways and parameterized such that it will always operate in a biologically plausible range.

We also adjusted the model descriptions in the Methods section, and. For example, for the PEIRS model we now write

$$p_i = \frac{exp(\beta(Q_i + tanh(\omega\delta_{stimulus})\, S_i))}{\sum_j exp(\beta(Q_j + tanh(\omega\delta_{stimulus})\, S_j))}$$

to include the constraint.

[1] Möller, M., & Bogacz, R. (2019). Learning the payoffs and costs of actions. PLoS computational biology, 15(2), e1006285.

2 The Prediction Errors Induce Risk Seeking (PEIRS) model is presented as the most

parsimonious model for describing how participants modulate risk seeking as a function of stimulus prediction errors. I agree with the authors that this seems to be the only model that could capture both the risk preference when δ_stimulus>0 and the risk aversion when δ_stimulus<0. However, the core assumption of this model, that δ_stimulus=mean(shown (Q))-mean (all (Q)), makes the prediction that there should be negative prediction errors for reward-predicting stimuli. There are numerous examples in the literature (Tobler, Fiorillo, Schultz 2005 as one compelling example, 10.1126/science.1105370) that show that dopamine neurons increase firing in anticipation of reward-predicting cues, even if less than the average over all stimuli. Given the neurally-inspired origins of the authors' theory, it seems that PEIRS likely must be rejected on the basis of extant neural recordings.

The reviewer raises an important point that we very much should have discussed further in our manuscript. The reviewer rightly points out that according to the PEIRS model, we would expect to observe lower than baseline DA activity after a negative stimulus prediction error. This implication of the theory seems at first glance to be contradicted by some empirical findings. For example, as the reviewer points out, (Tobler, 2005) finds that DA activity increases, even if a stimulus is associated with below average reward, as shown below:

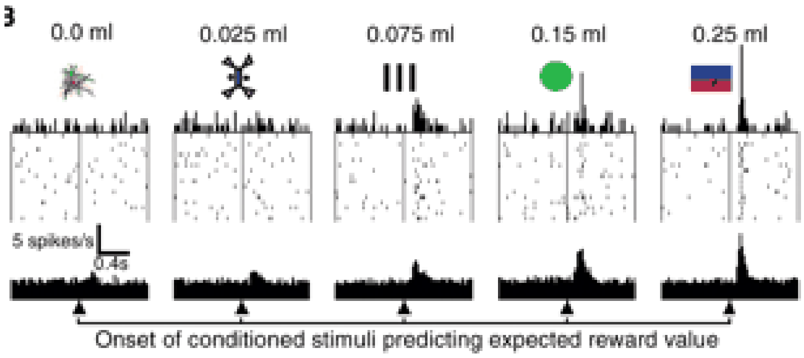

However, we believe these findings are in line with our theory. First, note that in the above figure there is even some small initial above baseline DA activity for the stimulus not associated with any reward. This rise in activity is likely because Tobler et al. employed a variable ITI. As such, the (unexpected) appearance of a stimulus on screen, which on average is associated with reward, is an event that evokes a positive prediction error. Once stimulus identity has been established (which likely happens in the order of milliseconds after stimulus onset), this initial positive response is modulated by the

reward associated with the particular stimulus. This task-design conflates the prediction error associated with the beginning of a trial with what we call the stimulus prediction error (the difference between the average stimuli value and the stimuli value on a trial).

In contrast, in our task design we employ a fixed ITI, which dissociates these two prediction errors, with one occurring at the beginning of the ITI and the other at the end of the ITI. An example of a similar task with a fixed ITI during which midbrain dopamine was recorded is [1]. Here, the authors employed a fixed ITI of 400 ms and, in one condition, also presented stimuli that were associated with different reward values.

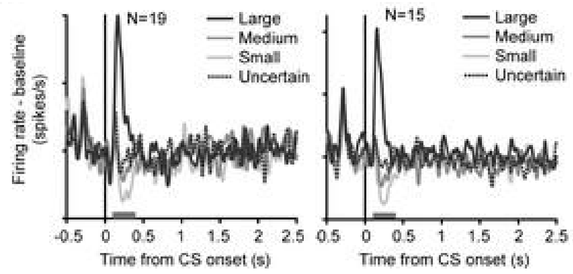

As can be seen from the above figure, the authors observe an initial spike in DA activity 400 ms before CS onset, which is associated with the unexpected beginning of a trial, and then another burst of activity shortly after the stimulus identity is revealed. Here it becomes clear that while the initial burst of activity 400 ms before CS is always positive, lower than baseline firing is observed if the stimulus predicts a lower than average reward (small).

To avoid any future confusion, we have adapted our theory section to emphasize the timing aspect of the trial structure. The manuscript now reads

> At the beginning of a trial (before the stimuli appear), participants do not have any specific information to base their prediction on. Given that our task design contains a fixed ITI and trials have the same structure throughout, we may assume that participants anticipate the appearance of stimuli at a certain time after the initial fixation. The corresponding reward prediction at that time should then be an average

over all possibilities, i.e., an average over the learned values of all options that might occur.

We have further added paragraphs to the discussion, to discuss the issue in depth.

Our theory rests on the occurrence of stimulus prediction errors to explain risk preferences. Generally speaking, positive stimulus prediction errors would explain risk-seeking, while negative prediction errors would explain risk-aversion. We indeed observe risk-seeking as well as risk-aversion (see Fig 2C) and find that those effects are best explained by the PEIRS model, which features positive as well as negative prediction errors (see Fig 3). While this explanation is consistent in itself and compatible with the data we collected, one might question its biological plausibility, on the grounds that reward predicting stimuli are known to elicit dopamine bursts, but not dips. For example, a classical study shows increased dopamine activity as a response to reward-predicting stimuli, even for stimuli that predict a relatively small reward (Tobler, Fiorillo et al. 2005).

There seems to be a contradiction between these results and our assumptions, but they are in fact compatible. To see this, one must consider the details of the trial structure in the study in question: while our study had a fixed ITI, many classic studies (such as (Tobler, Fiorillo et al. 2005)) have a variable, random ITI. This means that while our participants could predict the time of stimulus onset perfectly, the onset was surprising for the subjects in (Tobler, Fiorillo et al. 2005). This is a crucial difference: if the time of stimulus presentation can be predicted, a reward expectation for that moment can be formed, and the value of the stimuli can be compared to this expectation. If the time of stimulus onset is unknown, then any stimulus that appears will first and foremost be compared to the possibility that nothing happens in that moment. The result of that comparison must be positive, as the occurrence of reward predictors is always better than their absence.

This difference is manifest in neural recordings. For example, fixed ITIs were used in combination with stimuli that predicted different reward sizes in (Wang, Toyoshima et al. 2021). The dopamine activity shown in Fig 2E and 2F of that study clearly dips below the baseline level for stimuli that predict relatively small rewards. This suggests that the assumptions of the PEIRS model are biologically plausible for tasks with fixed ITIs, such as the one we are using in this study.

We did nevertheless include a model based on reward predictions, not prediction errors, which assumes dopamine responses like those in (Tobler, Fiorillo et al. 2005). That was the PIRS (Predictions Induce Risk Seeking) model, which we included in our model comparison (Fig 3D). Simulations are shown in Fig S5. Both simulations and model comparison show clearly that PIRS does not explain the data well.

In summary, we may conclude that prediction errors, but not predictions, might induce risk-seeking in our task, and that the underlying mechanisms are consistent with what is known about dopamine release in tasks with predictable timing.

[1] Wang, Y., Toyoshima, O., Kunimatsu, J., Yamada, H., & Matsumoto, M. (2021). Tonic firing mode of midbrain dopamine neurons continuously tracks reward values changing moment-by-moment. Elife, 10, e63166.

3 The authors present the Predictions Induce Risk Seeking (PIRS) model as an alternative to PEIRS. The δ_stimulus for PIRS is strictly positive in this task (lines 215-216), which accords well with neural data. This model is able to capture the behavioural data in Figure 2C, and shows stronger risk preference for the high-value stimuli than for the low-value stimuli. However, this model predicts risk-seeking for all δ_stimulus>0, which seems to run directly counter to the behavioural results shown in Figure 2B. Given a clear behavioural effect, I would argue that these data falsify the model.

The reviewer points out that the PIRS model makes predictions that are not supported by our data. This is further corroborated by the additional analysis prompted by the

reviewer's next question (see below). We strongly agree with the reviewer that our data provides more evidence for PEIRS than for PIRS, and we should have deemphasised PIRS in our manuscript.

We adjusted our paper accordingly to focus on PEIRS as the main model. This represents our results more clearly--prediction error induced risk seeking but NOT prediction induced risk seeking might explain risk preferences in learning tasks.

To further clean up our manuscript, we now present the detailed simulations of some models we tested as supporting information instead of the main text. We also moved the simulations of the PIRS to this supplementary figure but still mention it in the Results section. We also kept the details of the PIRS model in the Methods section, and emphasise that the evidence supports PEIRS more than PIRS in the Discussion. The revised Discussion now reads:

> We did nevertheless include a model based on reward predictions, not prediction errors, which assumes dopamine responses like those in (Tobler, Fiorillo et al. 2005). That was the PIRS (Predictions Induce Risk Seeking) model, which we included in our model comparison (Fig 3D). Simulations are shown in Fig S5. Both simulations and model comparison show clearly that PIRS does not explain the data well.
>
> In summary, we may conclude that prediction errors, but not predictions, might induce risk-seeking in our task, and that the underlying mechanisms are consistent with what is known about dopamine release in tasks with predictable timing.

4 Why only simulate the risk preference data in Figure 2C? The authors mention several times that a key advantage of their approach is to simulate trial-by-trial data (e.g. lines 581-583) yet they only show the summary statistic for risk preferences. The simulations would strongly benefit from trial-by-trial analysis to show that the best models also capture the learning effects in Figures 2A,B. As mentioned above, this analysis would have likely falsified PIRS.

The reviewer suggests that our approach would allow for a more detailed analysis of trial-by-trial data: we could use the models to simulate how risk preferences arise over the course of learning. These curves could then be compared to the empirical results we show in Fig 2, allowing us to test our models more thoroughly, and potentially falsify several.

We think this is an excellent idea, and have carried out the suggested analysis as proposed. The results are shown in Fig 3B for four of the models and in Fig S5C for all other models. The PIRS panel in Fig S5C suggests that the reviewer's intuition about PIRS was correct—as suggested, it is falsified by these simulations.

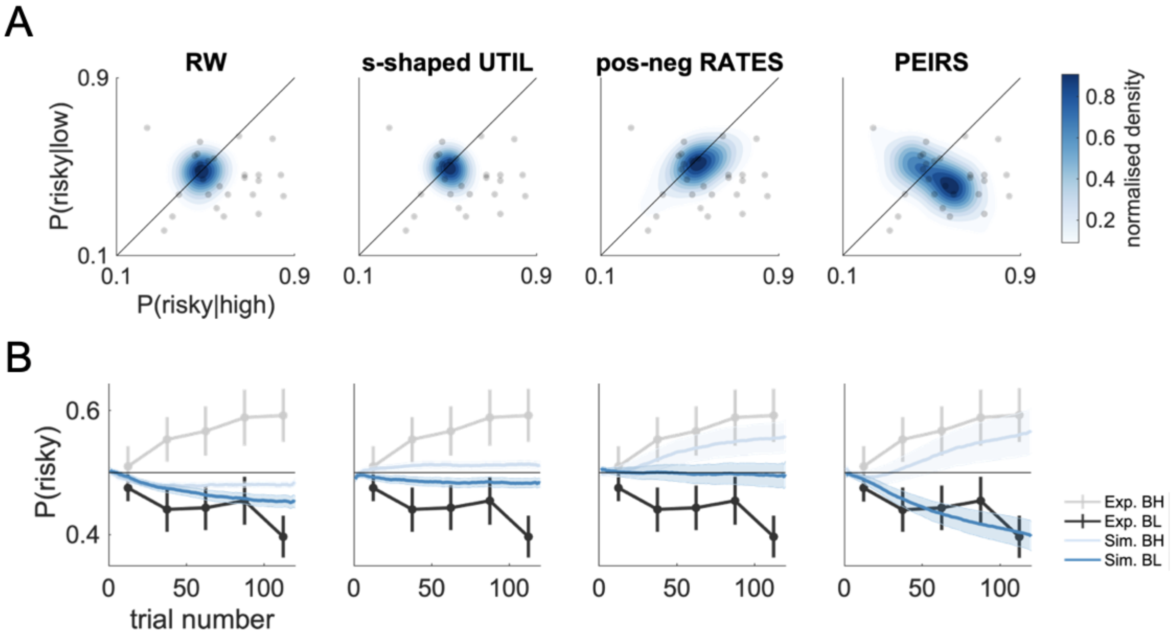

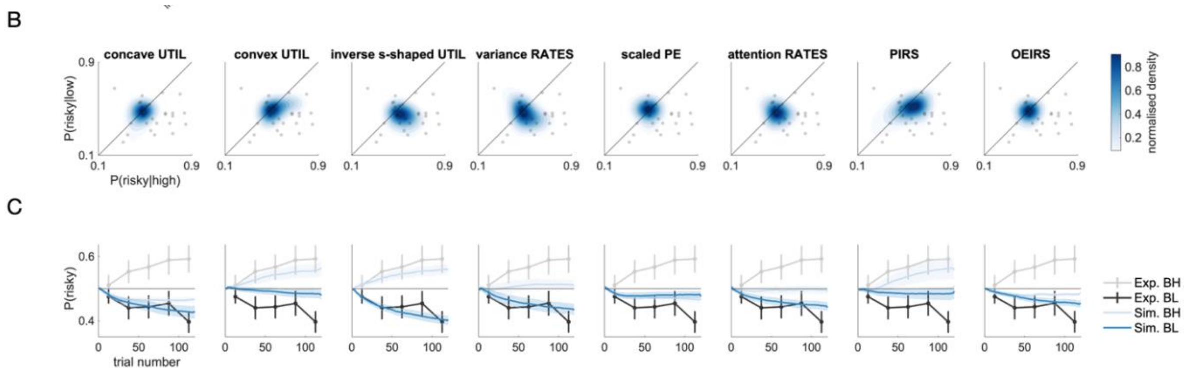

The figure legend reads

B) The temporal evolution of risk-preferences in simulated datasets. Risk preferences were extracted from simulated (Sim.) datasets, split between conditions (BH: Both-High, BL: Both-Low) and plotted as a function of trial number (light blue for the both-high condition, dark blue for the both-low condition). The shaded areas indicate the SE across simulated participants. The simulated data was smoothed using a moving average. The corresponding experimental (Exp.) data is superimposed in grey (light grey for the both-high condition, dark grey for the both-low condition), represented in the same way as in Fig 2C.

The Methods section outlines the procedure as

For Fig 3B, we first split the risk preferences between conditions. We then averaged the traces over repetitions of the experiment for each simulated participant, and finally over participants. For display, the averaged simulated traces where smoothed with a 20-point moving average filter.

In the discussion we also point to these simulations as further evidence for PEIRS:

Finally, we used trial-by-trial modelling to test the mechanism that we propose, alongside various alternative explanations. A combination of simulations and model selection techniques revealed that out of all tested models, our hypothesis is the most likely explanation for the effects that we observed.

5 Assuming that the computational form of PEIRS is correct (though, as I mentioned above, I do not think the neural implementation would correspond to real neural data), the model makes the prediction that when δ_stimulus=0, there should be no risk preference. This is a clear prediction of the model that should be tested.

When predicting risky choices from the stimulus PE in a logistic regression (including a random intercept for each participant and also allowing the stimulus PE to vary as a random slope for each participant) we find that the overall intercept across participants is not significant (likelihood ratio test, p=0.317, X2(1)=1.002). This is in line with the model prediction the reviewer suggests to examine.

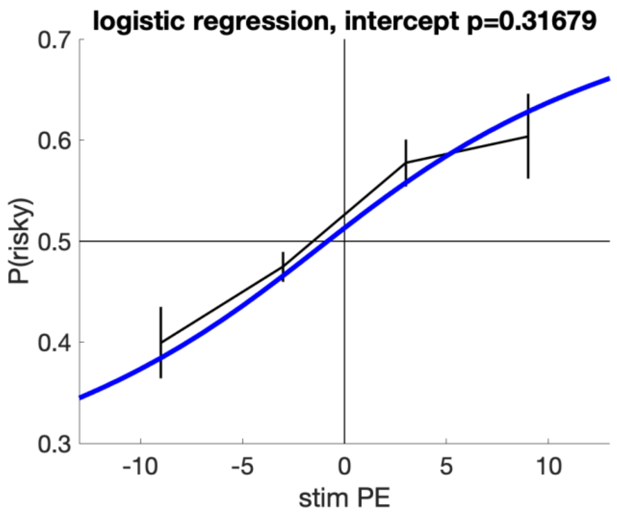

However, the analysis provided here is somewhat complicated: it mixes empirical variables (P(risky)) with latent variables estimated through model fits (stim PE). An additional model is then required to test the relationship. Due to this complexity, and the substantial length of the manuscript as is, we decided to not include the figure.

6 Support for the PEIRS model would be strengthened dramatically if the authors can show that risk preferences for the exact same stimuli vary as a function of δ_stimulus. As one example, imagine a task with six stimuli – risky-high, safe-high, risky-medium, safe-medium, risky-low, safe-low. On a particular trial, subjects can be cued that the possible set of stimuli for that trial will be drawn from risky-high, safe-high, risky-medium, safe-medium. This would set the baseline for comparison. Then, subjects would be given risky-medium and safe-medium as options on that trial. This would elicit δ_stimulus<0, and the theory predicts that participants would choose safe-medium. If, however, the set of possible stimuli for that trial were risky-medium, safe-medium, risky-low, and safe-low, and subjects were given risky-medium and safe-medium as options, this would elicit δ_stimulus>0, and the theory would predict that participants should choose risky-medium. Though this particular task variant may

not be feasible, a task that tests this idea would strongly support the idea that δ_stimulus modulates risk preferences.

We agree with the reviewer that tweaks to the task, such as the one described here, would greatly strengthen our claim. The idea of cuing different baselines would indeed allow us to experimentally manipulate the stimulus PE for the same set of stimuli. Given the time constraints for these revisions we are unfortunately not able to run such a task now. We also believe they would be beyond the scope of one manuscript, and given the space needed to develop the computational model. However, in future work we would indeed like to include such manipulations. We included the reviewer's idea in the discussion, which reads

> Other, more elaborate tests on the level of behavior are possible. For example, one might increase the set of stimuli and indicate a subset of available stimuli before the trial begins. The primed subset should affect expectations and hence stimulus prediction errors. With such manipulations, one might be able to induce opposite risk preferences in the same set of options, depending on the primed subset.

7 Given the trial-by-trial nature of the PEIRS model, do the authors have the power to assess trial-by-trial changes in risk preference as a function of updates to S? In other words, how do risk preferences evolve following large updates to S vs small updates (or decreases) to S? Though I suspect the authors may be underpowered for this analysis, this is another prediction of the theory.

The reviewer points to a potential link between updates in S and changes in risk preference. While conditions may exist under which such a link might be observed, we find it quite difficult to make a prediction how the two quantities would be linked in general in our experiment.

For example, consider a case in which all the Q's and S values are at their optimal values (i.e. reflecting ground truth). Now the subject chooses stimulus i and experiences

a reward that increases Si. Note that this could be either a very low reward (making the respective Qi go down) or a very high reward (making the respective Qi go up).

Let us now assume that the chosen stimulus occurs again on the next trial. What has changed? The update has increased Si but also increased or decreased Qi and hence the stimulus prediction error. All three variables contribute to the choice. In addition, whether or not the risky stimulus is chosen will depend on the S and Q of the other stimulus as well. We see that a mere increase in S of one option might be followed by increased risk seeking or decreased risk seeking, depending on several other factors.

Overall, it appears to us that the relationship between risk-seeking, available options on successive trials, risk updates, value updates and stimulus prediction errors is highly complex and does not seem to allow for many simple predictions other than the ones we already included. The proposed analysis seems intriguing, but we don't see how it is feasible with the data at hand. However, if we misunderstood what analysis the reviewer suggested than we are happy to stand corrected.

8 Regarding the pupillometric data, I have two questions/concerns. First, the authors should include other relevant regressors in their models (e.g. stimulus identity, stimulus chosen, relevant latent variables) and see how much their results change.

For various reasons, we decided to reorganise our pupil analysis and to remove it from the main text (see also Point 9 below). The analysis is now much less prominent. Still, we revised it, now controlling for the identity of the chosen and the unchosen stimulus (or, equivalently, for stimuli identity and stimulus chosen), among other changes.

While our results changed slightly in shape, they did not change qualitatively, i.e., we still find significant responses to the magnitudes of both prediction errors. The new figure showing the time courses is reproduced below:

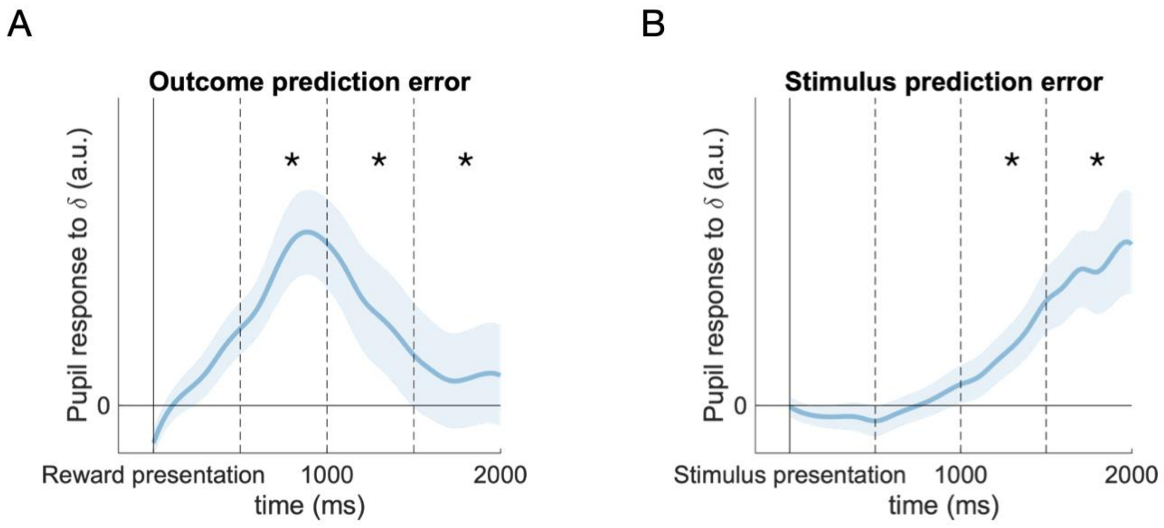

The details of our analysis are described in supporting information. There we now write

> We further included the stimulus identity—i.e., the fractal picture that was used—of the chosen and the unchosen stimulus as control regressors.

9 Second, shouldn't the pupil-behaviour comparison for Figure 4C be a comparison between pupil response and strength of risk preference? I would expect those participants with the strongest pupillometric responses (and therefore, the strongest encoding of δ_stimulus) would show the strongest risk preferences (and risk preference asymmetry, as according to the model).

After careful consideration of the reviewer's comments, and also the comments from the other two reviewers, we have decided to remove the pupil analysis from the main text and only present it as supporting information, in a very reduced form. The comparison that the reviewer mentioned is not any more part of our manuscript.

The reason for this change is that while we initially thought that presenting this data would strengthen the claims in our paper, we now feel that the pupil analysis adds unnecessary complexity and distracts from the modelling work that is the main focus of this article (as PLoS C.B. is a computational journal). Given that pupil dilation indexes noradrenaline and not dopamine (as reviewer 3 also pointed out), which is not

part of our computational model, it seemed to add unnecessary confusion about the nature of the physiological variables involved.

Minor comments

M1 Eq. 3 appears miswritten – as is, S would always increase. I imagine the authors meant to put Eq. 12 in its place.

The reviewer spotted a critical typo. We have fixed Eq. 3, which is indeed identical to Eq. 26 in the revised manuscript (former Eq. 12).

M2 Figure 2C – at what time points are the risk preferences being measured? I would assume the latter half of the session, when choices and risk preferences have stabilized, though this needs to be reported.

The risk preferences shown in Fig 2C were in fact measured across all trials, not just the latter half. We clarified this in the text, which now reads

> Correlation between risk preferences in the both-high mean and the both-low condition across all trials.

M3 The predictions in lines 250-251 do not match with order of predictions in lines 289, 301.

We were indeed inconsistent with our numbering. We swapped the predictions in lines 289, 301, making the Behaviour section consistent with the Theory section. We also changed the order of the Panels in Fig 2 (Fig 2B became Fig 2C, and vice versa).

M4 In line 308, the authors state "This is consistent with our theory: the first effect rests on more assumptions than the second" It is not clear how this follows – an effect resting on more assumptions does not guarantee a weaker effect size.

We agree that this paragraph was more confusing than helpful. We hence removed it from the manuscript.

M5 Eq. 10 - δ_coutcome should be δ_outcome

We corrected the typo.

M6 In line 662, authors state "Utilities can also be s-shaped, with different curvatures on both sides of a reference point." However, the proposed utility function is symmetric on both sides of 0.

We see that our sentence is ambiguous. What we meant to say is that there are utility functions with negative curvature for smaller values and positive curvature for higher values. The magnitude of the curvature might be the same on both sides. We rephrased that sentence to make it clearer. It now reads

> Utilities can also be s-shaped, with different signs of curvature on both sides of a reference point (Kahneman and Tversky 2013)—concave for values below the reference point, and convex for values above.

M7 In line 666: U = m + s, should be written as U = m + sigma

We corrected the typo.

M8 The following statements are far too strong for what is shown, as showing a significant association does not prove the existence of that signal (e.g., unmeasured confounders, etc…) Line 436: "we prove the occurrence of stimulus prediction errors by extracting the corresponding pupil response." Lien 475: "This confirms the existence of a stimulus prediction error that occurs after stimulus onset, as hypothesized."

We agree with the reviewer that our phrasing was stronger than the results would support. Both statements became superfluous with our reorganisation of the pupil results. In the new description, we made an effort to discuss the results more conservatively.

M9 In estimating the pupillometric stimulus prediction error signal, the authors censored the pupil recordings after reward presentation. It seems that the pupil signal contains time periods corresponding to choices. What is the effect of choice on the pupil signal and could that affect the interpretation?

The reviewer raises an important point: choice might be a confound for the pupil response to the stimulus prediction error. We have tested this by censoring all pupil data after choice, and computing the resulting trace:

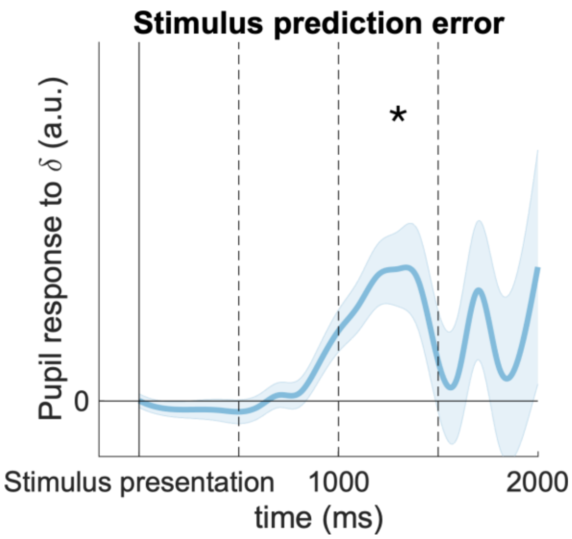

We find that the first part of the trace (up to about 1300 ms) does not change much as compared to the trace obtained without censoring:

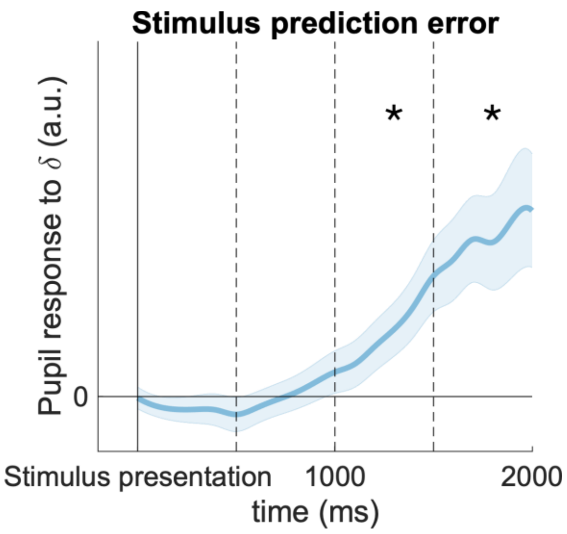

In particular, we still find a significant response to the prediction error magnitude. After 1300ms, the trace becomes increasingly noisy. This is likely due to the fact that most choices happen earlier (to be exact, 75 % of choices happen within 1.1946 ms after stimulus onset).

We conclude from this analysis that choice does not affect our results dramatically, and that we do not need to censor it. However, this was an important check.

M10 Describe the cluster-based permutation test in the methods.

The reorganised pupil section does not any more include the cluster-based method. Instead we used a simpler statistical test which is described in the supplementary methods as

> To test whether the pupil responses to the prediction errors are statistically significant, we followed (Browning, Behrens et al. 2015): we first split the pupil traces in four sequential 500 ms time bins. We then conducted a one-tailed t-test in each of these bins and applied the Bonferroni correction for multiple comparison to the results of the individual tests.

M11 Q_0 is a fixed parameter, set to 50. How would model fits change if Q_0 was a free parameter or if it was set to 0? S_0 is set as a free parameter, so it seems worthwhile exploring the effects of Q_0 being free as well.

The reviewer asks why Q_0 is not a free parameter, and what would happen if it was. Initially, we had indeed included Q_0 as a free parameter in all models. We found, though, that this allowed too much flexibility, which led to overfitting.

Consider for example the RW model, and assume that all initial values are 0. The task starts, participants start making choices. Empirically, we know that they choose risky-high more often than save-high, and save-low more often than risky-low.

Now, because the initial values are 0, all outcome prediction errors will be positive for many trials. In this phase, risky-high and the save-low will acquire more value than save-high and risky-low, just because they are chosen more often. This is how the RW model can fit the risk preferences without actually explaining them.

Such spurious explanations can be avoided by fixing the initial values at 50 points. Additional support for this assumption arises from the fact that participants will go through a few training trials before the main task starts. In those, they experience rewards around 50 points, which will frame and anchor their expectations in that range. Note that for S_0, there is no obvious initial value--we hence treat it as a free parameter to avoid bias.

M12 Figure 4C needs units.

Figure 4C has been removed from the manuscript as part of the revisions.

Reviewer #2

In the manuscript titled "A Behavioural Association Between Prediction Errors and Risk-Seeking: Theory and Evidence", Moeller and Colleagues use computational approaches alongside behavioural experiments to investigate the effect reward prediction errors (RPEs) have on risk preferences. Specifically, authors hypothesize that when learning about risky options (e.g., options with reward magnitudes that are drawn from different distributions), choice behaviour is modulated by stimuli prediction errors (how much the average value of the presented stimuli differ from the average value of all stimuli). Authors then used a simple arm bandit task to investigate the predictions of their hypothesis.

I find the question proposed in this paper is of value. However, I have major concerns about the strength of the results presented and the analysis performed, which needs to be addressed. Additionally, the writing needs to improve dramatically, the paper is not very well written and difficult to read (some suggestions have been provided below).

> Thank you for your suggestions below. We feel that the additional analyses you suggested indeed strengthen our conclusions, and we hope that we were able to adequately address your concerns. Finally, we apologise for the sloppy writing—we have adjusted the manuscript according to your comments and also changed the writing in some other places.

Major concerns

1) Page 2, line 18: Authors don't talk about their hypothesis/theory until line 21 and even then, it is not clear what their hypothesis is. Please revise the abstract and clarify what is being tested/hypothesized.

> We agree that our abstract was not as precise and effective as it could be, especially with respect to stating a hypothesis. We adapted the wording to make sure that our hypothesis is stated clearly and early on. We further spelled out the central prediction that is tested. We hope that the abstract is clearer now. It reads:

Reward prediction errors (RPEs) and risk preferences have two things in common: both can shape decision making behavior, and both are commonly associated with dopamine. RPEs drive value learning and are thought to be represented in the phasic release of striatal dopamine. Risk preferences bias choices towards or away from uncertainty; they can be manipulated with drugs that target the dopaminergic system. Based on the common neural substrate, we hypothesize that RPEs and risk preferences are linked on the level of behavior as well. Here, we develop this hypothesis theoretically and test it empirically. First, we apply a recent theory of learning in the basal ganglia to predict how RPEs influence risk preferences. We find that positive RPEs should cause increased risk-seeking, while negative RPEs should cause risk-aversion. We then test our behavioral predictions using a novel bandit task in which value and risk vary independently across options. Critically, conditions are included where options vary in risk but are matched for value. We find that our prediction was correct: subjects become more risk-seeking if choices are preceded by positive RPEs, and more risk-averse if choices are preceded by negative RPEs. These findings cannot be explained by other known effects, such as nonlinear utility curves or dynamic learning rates.

2) Authors only include concave and s-shaped utility functions in their analysis. However, it seems to me that including a convex or inverted s-shaped utility function can explain parts of the reported results. Convex utility functions have been reported in non-human primates [1].

The reviewer suggests that in addition to concave and the s-shaped utility, we should also consider convex utility and inverse s-shaped utility as alternative explanations. We agree that these utility functions might also be of interest to some readers of our manuscript. While somewhat more obscure than concave and s-shaped utility, it is interesting to check how they might be able to explain our data.

We hence constructed the corresponding models, and included them into our set of candidate models. We report them in the same way as the other secondary alternative explanations (i.e., they are included in Fig 3D, the complete model comparison, and in Fig S5, the additional simulations). We found that convex utility (described in monkeys in [1], as pointed out by the reviewer) is not a good description of our data: it produces the wrong risk preference pattern in simulations and loses the pairwise model comparison. Inverse s-shaped utility (not normally used in neuroeconomics, at least to our knowledge) could reproduce the observed risk preferences in simulations, but lost against PEIRS in a direct model comparison when fitted to our data.

These results suggest that concave as well as inverse s-shaped utility can be discarded as alternative explanations. Concave utility was falsified directly; inverse s-shaped utility can accommodate the risk preference patterns, but it does not explain them as well as PEIRS, which is favoured in the model comparison. This is despite PEIRS having more free parameters for which it gets penalised in the model comparison. Our model recovery analysis (see below) suggests that this result is dependable, as it is unlikely that those two models should be confused.

From these additional analysis, we conclude that neither of the two proposed utility functions explains the effect in our data as well as the explanation we propose. In addition to this empirical reason, we think that there are also fundamental reasons why convex and inverse s-shaped utility functions should not be considered valid alternative explanations, even if they weren't falsified and fitted better than other models.

First, let us consider the level of explanation: utility functions can be used to capture behavioural effects, i.e., they can provide a compact description of certain aspects of behaviour (such as risk seeking). Such models might well be used to make predictions about behaviour. What they cannot provide us with, however, is an explanation on the level of neural processes (a distinction phrased as 'aggregate' versus 'mechanistic' in [B]). In fact, it might well be that for a given neural process, one may find a utility function that can compactly summarize the effects it has on behaviour. These two descriptions are placed on different levels, and comparing them might not be meaningful [B].

Second, let us consider generalisability: concave and s-shaped utilities are well documented and embedded in broader theories (expected utility theory and prospect theory, respectively). The effects that they describe appear in many different situations, they seem to capture a fairly general aspect of behaviour (which perhaps relates to a general mechanism in the brain). In contrast, convex and inverse s-shaped utility functions only describe behaviour in some very specific tasks (as in the one the reviewer referenced), but fail to generalize to others. It appears as if one is overfitting the concept of utility, at the price of specificity--everything can be explained as an effect of non-linear utility, but one needs a different function for each situation. This does not seem a desirable state. From the standpoint of generality, it seems appropriate to test established theories such as prospect theory, but problematic to include utility functions tailored to the behavioural effect in question.

In summary, we tested the proposed models, and found that the proposed utility functions do not explain our effect as well as the PEIRS hypothesis. We also see epistemological issues with non-standard utility functions, and hence include the models as secondary explanations. We included a section with these arguments in the Discussion section, and discuss the empirical results of the two additional models as appropriate. Details of the utility functions have been added to the Methods sections.

The figures showing the results for convex and inverse s-shaped utility are shown below:

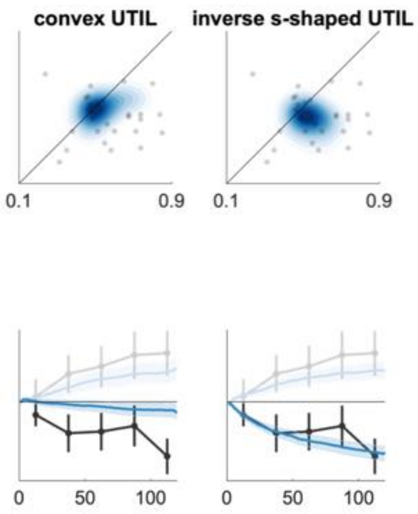

Our Discussion now reads as follows:

Relation to utility models

Several of the models we have tested are based on nonlinear utility of rewards. The central idea of these models is that participants might not find an outcome of 100 points twice as rewarding as an outcome of 50 points. If the perception of reward is distorted in this way, risk preferences might arise as a consequence (Kahneman and Tversky 2013). None of the tested utility models constituted a better explanation of our effects of interest. However, one of them (inverse s-shaped UTIL) at least reproduced the trial-by-trial emergence of risk preferences well (Fig S5C). How should this and the other utility-related results be interpreted? We see two issues with utility models.

The first issue relates to the level of explanation: utility functions can be used to capture behavioral effects, i.e., they can provide a compact description of certain aspects of behavior (such as risk-seeking). Such models might well be used to make predictions about behavior. What they cannot provide us with, however, is an explanation on the level of neural processes (this distinction was phrased as 'aggregate' versus 'mechanistic' in (Palminteri, Wyart et al. 2017)). In fact, it might well be that for a given neural process, one may find a utility function that can compactly summarize the effects it has on behavior. These two descriptions then concern different levels of description, and comparing them might not be meaningful (Palminteri, Wyart et al. 2017). In this study, we derive a mechanistic model (PEIRS), and show that it is the best explanation of the effects of interest. Our level of description is thus the mechanistic one.

The second issue is generalizability and affects convex and inverse s-shaped utility functions in particular. Concave and s-shaped utilities are well documented and embedded in broader theories (expected utility theory and prospect theory,

respectively). The effects that they describe appear in many different situations—they seem to capture a fairly general aspect of behavior (which perhaps relates to a general mechanism in the brain). In contrast, convex and inverse s-shaped utility functions only describe behavior in some very specific tasks (see (Stauffer, Lak et al. 2015) for behavior that is well described by convex utility) but fail to generalize to others. In these cases, one might be overfitting the concept of utility, at the price of specificity. At the extreme, it is conceivable that most phenomena can be explained as an effect of non-linear utility but require a specific (and perhaps quite non-trivial) utility function for each case. From the standpoint of generalizability, it seems thus seems appropriate to test established theories such as prospect theory as serious alternative explanations. Utility functions tailored to the behavioral effect in question seem more problematic.

Overall, we find that neither our empirical results nor general epistemological considerations indicate that much emphasis should be put on utility models in the context of our task and our goal (to understand the neural mechanisms that cause risk preferences).

[A] Zalocusky, K. A., Ramakrishnan, C., Lerner, T. N., Davidson, T. J., Knutson, B., & Deisseroth, K. (2016). Nucleus accumbens D2R cells signal prior outcomes and control risky decision-making. Nature, 531(7596), 642-646.
[B] Palminteri, S., Wyart, V., & Koechlin, E. (2017). The importance of falsification in computational cognitive modeling. Trends in cognitive sciences, 21(6), 425-433.

3) Similarly, models that allow for scaling of RPEs ([2-3]), may be able to explain the observed behaviors and should be tested.

The reviewer suggests that scaled prediction error models might constitute an alternative explanation for our effects. This is an intriguing idea—those models are related to the ones we study here, as they also track the variability of reward.

We created a scaled model within our framework, and tested in the same way as we tested the other models. The results are reported in the same way as the other secondary alternative explanations (i.e., they are included in Fig 3D (complete model comparison) and Fig S5 (additional simulations)).

We find that the fitted scaled prediction error model does not reproduce the risk preferences of interest, and also loses against PEIRS in the model comparison. Overall, we find that while the scaled prediction error model is a very interesting idea, our dataset does not support it. Details of the specific model we tested can be found in the Methods section. There we write

Scaled prediction errors

Several studies have indicated that dopamine signaling (and hence reward prediction errors) might adapt to the magnitude of reward fluctuations (Tobler, Fiorillo et al. 2005, Diederen and Schultz 2015, Diederen, Spencer et al. 2016). It is thought that the adaptation is achieved through scaling reward prediction errors relative to the perceived reward variability, which is tracked alongside the average reward. Such scaling might affect stimulus-specific learning speeds and could hence introduce risk-related choice biases.

Here, we model the scaled prediction error hypothesis by implementing scaled prediction errors on top of the model of (Mikhael and Bogacz 2016). The model is then defined as

$$\tilde{\delta}_{outcome} = \frac{r - Q_{chosen}}{S_{chosen}}$$

Eq. 30

$$\Delta Q_{chosen} = \alpha_Q \times \tilde{\delta}_{outcome}$$

Eq. 31

$$\Delta S_{chosen} = \alpha_S \times (|\tilde{\delta}_{outcome}| - 1)$$

Eq. 32

Choices are modeled using the softmax rule Eq. 15. The scaled prediction error model has free parameters $\alpha_Q \in [0,1]$, $\alpha_S \in [0,1]$, $S_0 > 0$, $\beta > 0$, and fixed parameters $Q_0 = 50$.

4) Authors use the first 60 trials when calculating the correlation between pupil dilation and outcome prediction errors, while they use the last 60 trials for calculating the correlation between pupil dilation and stimulus prediction error. If the fact that learning has been accomplished by 60 trials means that the RPE doesn't exist (which is not a correct assumption), then it doesn't make sense to include final 60 trials for outcome prediction error. What will happen if all trials are included in both analyses?

The reviewer questions our assumption regarding the trials we include in our pupil analysis and rightly points out that RPEs still occur even if participants have fully learned the task. We apologise for not including a proper discussion of why we only focused on certain trials throughout the task for our pupil analysis. The reason we focused only on certain trials is that pupil dilation does not signal RPEs but a related quantity that scales with the amount of information that is gained on a trial [A]. During learning this information gain is correlated with (unsigned) RPEs but this is not the case anymore once learning has finished.

Nevertheless, for the sake of simplicity, we have adapted our analysis, and now include all 120 trials for both prediction errors. We have reorganised the pupil section significantly; the results that we keep are not affected substantially by this change (the shape of the stimulus prediction error changed slightly, but both prediction errors can still be clearly detected).

[A] Zénon, A. (2019). Eye pupil signals information gain. Proceedings of the Royal Society B, 286(1911), 20191593.

5) Page 22, Fig. 4C: it seems that there are two types of subjects, those whose value for (BIC_rw – BIC_peirs) are negative (adopted RW model) and those whose value for (BIC_rw – BIC_peirs) are positive (adopted PEIRS model). Surprisingly, the majority of subjects' behavior is better explained by RW model. What will happen if percentage of subjects with smaller values of BIC are plotted in Fig. 3C, D. This figure suggests that among all subjects (30 of them), the behavior of only 7 of them was better explained by PEIRS model, which is driving the results in Fig. 3C, D, as well as the correlation in Fig. 4C.

The reviewer observes that the values on the x-axis in Fig 4C (orig. manuscript) imply that more participants seem to be better described by RW than PEIRS, and contrasts this with the results we show in Fig 3C: that the population is better described by PEIRS.

First, we want to point out that this is not a contradiction, but a result of the way our analysis is set up: by performing our model comparison on the population level, we implicitly assume that the entire population uses the same unknown model (with different parameters for different individuals) to generate data. Starting from that premise, our analysis then determines which of our candidate models is the most likely to be the unknown population model. The model recovery analysis (see below) indicates that our dataset supports a fairly accurate identification of the correct model, at least for those models that are most relevant. This means we can have some confidence that the population overall is better described by PEIRS, as per Fig 3D.

The idea of going further and identifying models on the level of single participants is indeed an intriguing one. Such analyses could provide us with relative frequencies of the models in a population. Unfortunately, our sample size is too small for this—the study was not designed for group level subdivisions and is hence underpowered for such analyses.

Note further that we have removed the figure in question (Fig 4C of the original manuscript) in the process of streamlining and deemphasizing our pupil analysis. Now, the reliability of single-subject modelling results does not affect any analysis we present in the new version of the manuscript.

Finally, we'd like to mention that our finding is supported by another recent study from our lab [C]. In that study, it was also found that PEIRS described the population better than RW. Moreover, in the dataset collected for that study, 23 out of 30 participants were better described by PEIRS (however, the same caveats apply about making population-level inferences). We included this reference in the manuscript, where we now write

> This is in line with new results that replicate our finding: (van Swieten, Bogacz et al. 2021) show that PEIRS describes their population better than RW, despite its increased complexity.

[C] van Swieten, M. M., Bogacz, R., & Manohar, S. G. (2021). Gambling on an empty stomach: Hunger modulates preferences for learned but not described risks. bioRxiv.

6) Authors use (BIC_rw – BIC_peirs) to quantify the prediction error induced risk preferences. Do authors have any evidence supporting this choice? For example, is there any correlation between the value of \gamma and the difference in BIC values? Then why not use \gamma values? Please clarify.

After careful consideration of the reviewer's comments, and also the comments from the other two reviewers, we have decided to remove the pupil analysis from the main text and only present it in the supplements, in a very reduced form. The analysis that the reviewer is referring to is not any more part of our manuscript.

Still, we'd like to clarify that gamma is not suitable to quantify risk-preferences: gamma is linked to measured variables (i.e., choices) only through a softmax function, and has substantial interactions with the softmax temperature beta, which in turn interacts with the learning rates alpha_Q and alpha_S. The absolute values of all of these parameters individually are thus not very meaningful quantities. The same is true for other measures such as differences in risk seeking, which was proposed by another reviewer: while these differences can be due to PEIRS, they might be amplified or modulated by other effects. All in all, the BIC difference were the clearest way to quantify PEIRS-specific risk preferences of an individual.

7) Comparison of likelihood of proposed models is used to provide strong evidence that PEIRS/PIRS variant of models can best explain the subjects' behavior. This begs the question of whether the models are well identifiable in this task. To test this, authors should simulate each model with a range of parameters, and fit the simulated behavior with each model, then show that each model fits better data simulated by the same model. This procedure also allows them to verify that the parameters are identifiable by the fitting procedure.

The reviewer requests a model recovery analysis to validate our model fitting analysis, and to back up the conclusions we draw from it. We fully agree that this is an important check, and have carried out the proposed analysis. We followed (Palminteri, S., Wyart, V., & Koechlin, E. (2017). The importance of falsification in computational cognitive modelling. Trends in cognitive sciences, 21(6), 425-433.), using parameters sampled from the empirical posterior distributions to check whether the models are identifiable for datasets similar to ours.

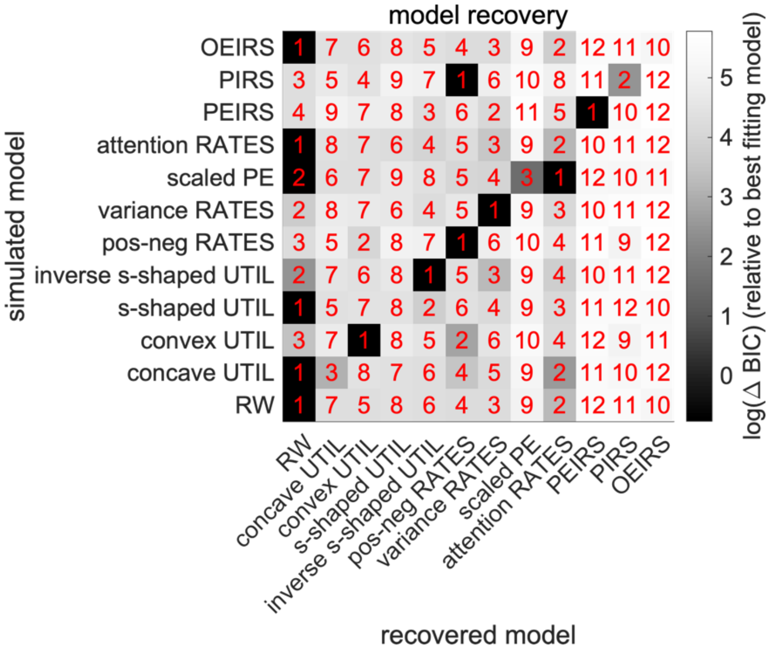

We added the analysis as a supplementary figure (Fig S6) and refer to it in the main text. The results suggest that the important models (especially PEIRS) are well identifiable. For some of the other models, RW is selected as a better description. This is not surprising or problematic--our task was not designed to identify many of the

effects we tested. Since those models are not confused for PEIRS or another model of primary interest, we may be confident that the outcomes of our analyses are dependable.

The text in our manuscript now reads

Model recovery

To validate our model selection procedures, we performed a model recovery analysis (Palminteri, Wyart et al. 2017). This analysis is meant to show whether our candidate models are identifiable in the empirical parameter range. We hence performed an a posteriori recovery analyses: first, models were fitted to the empirical dataset, which resulted in posteriors over the parameter space. Parameters were then sampled from those posterior distributions, and simulations were performed with the sampled parameters. Finally, all models were fitted to all simulated datasets, and model selection metrics (BIC) were extracted. This results in a confusion matrix. We repeated this procedure 100 times for each of our 27 participants (a total of 2700 repetitions) to account for stochastic fluctuations. The averaged confusion matrix is shown in Fig S6.

The confusion matrix suggests that 6 out of 12 models (RW, convex UTIL, inverse s-shaped UTIL, pos-neg RATES, variance RATES and PEIRS) can be directly identified by our procedure, with ground truth simulations based on the empirical parameter range. For another 5 out of 12 models (Concave UTIL, s-shaped UTIL, scaled PE, attention RATES, OEIRS), RW was selected as the better model to describe the datasets they generated. Given that all those models contain RW as a special case, this means that the additional effects they describe just do not occur in our dataset—their empirical parameter range is such that the models mimic RW and make no use of the additional features they can describe. The model comparison still penalizes the additional complexity, leading to RW as the selected model. The only model that is confused with a model other than RW is PIRS, which is confused with

pos-neg RATES. This means that for all models but PIRS, our model comparison will either select the correct model if an effect is there or the base model (RW) if the effect does not appear. Importantly, we find that in the empirical parameter range, PEIRS is selected unambiguously. This suggests that PEIRS being selected in the model comparison on the empirical dataset is a meaningful result.

Minor concerns

M1) Page 3, line 51-56: Please revise this sentence, it is very difficult to understand.

We split the sentence in several smaller sentences to improve readability. It now reads:

> The above-mentioned family of basal ganglia models includes these modulatory mechanisms too. This makes the models consistent with some well-studied phenomena whereby dopamine modulates how uncertainty and risk affect decision making. For example, dopaminergic medication can bias human decision making towards or away from risk (11-14). Further, phasic responses in dopaminergic brain areas predict people's moment-to-moment risk-preferences (15).

M2) Please provide a supplementary figure with average choice probability values for the other one condition (different).

We added a figure (Fig S3) that shows the probability of choosing the high valued stimulus over the low valued stimulus, split by stimuli combinations (there are four different pairings in the 'different' condition), and averaged over participants. We also included a pointer to the figure in the main text. The figure is reproduced below:

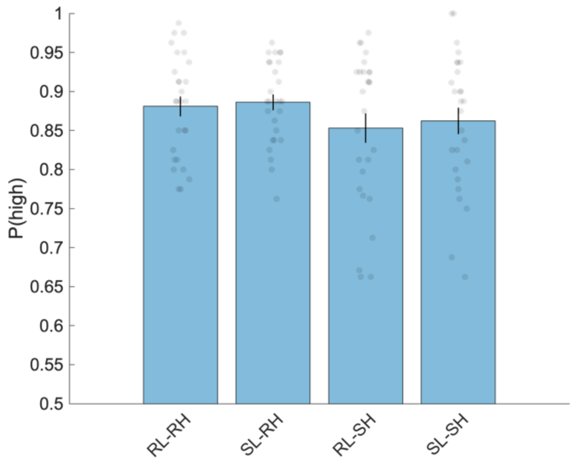

Fig S3: Choice probabilities in the different condition. We show the average probability of choosing the high-valued option, for all combinations of stimuli (RL: risky-low, SL: safe-low, RH: risky-high, SH: safe-high). The error bars indicate the SE of the mean across participants (N=27), the grey dots show individual participants.

M3) The performance cut-off value seems arbitrary, why 70% was chosen (Fig. S1 caption, however, says 65%)? Please clarify.

We corrected the inconsistency, changing the 70 % in the main text to 65 % as in the appendix (this was the value we used in the analysis).

This value was chosen to separate the participants into sensible clusters. In Fig S1, it can be seen that there are three participants that perform much worse than the others in the cohort. The value 65 % separates those non-learners from the well-performing participants, but is otherwise indeed arbitrary (70 % would have had the same effect).

We added this explanation to the figure to make our approach more transparent. It now reads

> To be included in our analysis, participants had to choose the high value option over the low value option at least in 65 % of the relevant trials across the experiment. This threshold was chosen to separate two clusters of participants that occurred in our

cohort—learners and non-learners (as can be seen in Fig S2, there are 3 subjects that perform substantially worse than the other 27).

M4) Please fix the following typos:

Page 22, line 458: …(trials 61-120).similar to A) (This time interval is not similar to A)

Typo was corrected.

Page 31, Models: Please add equation number to all formulas.

We have numbered all equations in the manuscript.

Page 2, line 36: … is precisely what our theory predicts …

Typo was corrected.

Page 12, line 244: … this also contributes to a higher (in) learning …

We corrected the typo.

Page 12, line 246: Relaxing (now) our assumption

Sentence was deleted for other reasons.

Page 31 line 627: … preferences emerged faster than risk preferences …

Sentence was deleted for other reasons (see minor point 9).

Page 33, line 678: Different learning rates for noisy and safe stimuli

We corrected the typo.

Page 34, line 692: … the spread ()of a reward signal …

Typo was corrected.

Page 34, line 693: … denotes (the spread) of the spread of the rewards

Typo was corrected.

Page 38, line 775: … against a trial-by-trial estimate …

Typo was corrected.

M5) Page 25, line 499: No proper motivation is given for the study. Please revise.

We revised the first paragraph of the discussion, now stating clearly the hypothesis we want to test. It reads

> Different behavioral phenomena—learning from prediction errors and biased risk-preferences–are attributed to the same neurotransmitter, dopamine. The common neural substrate led us to hypothesize that there might be an association between prediction errors and risk preferences on the level of behavior. To investigate this, we first used a theory of basal ganglia learning to develop a model that represents the mechanism that we propose, and to derive concrete predictions of the hypothesis.

M6) Page 25, line 504: … determines its effect on risk preferences. Using this/that/it/such is not very informative and should be avoided (the issue persists throughout the discussion section (e.g., line 517 and etc.)). Please revise.

The quoted sentence was deleted for other reasons. We have, however, revised our discussion, replacing references such as “this”, “it” etc. with more concrete expressions.

M7) Page 26, line 527: This information (exact numbers) should be provided in the results section.

We added the appropriate information in the results section and changed the discussion accordingly. We report that

Participants made their choices on average 0.97 s (standard deviation 0.51) after stimulus onset.

8) Page 28, line 587-8: This sentence is not written in a scientific tone. Please revise.

We reformulated the sentence, giving it a more neutral tone.

9) Page 31, line 626-627: This sentence belongs to results not methods section. Please remove it.

We removed the sentence.

References:

[1]. Stauffer, W. R., Lak, A., Bossaerts, P., & Schultz, W. (2015). Economic choices reveal probability distortion in macaque monkeys. Journal of Neuroscience, 35(7), 3146-3154.

[2]. Diederen, K. M., & Schultz, W. (2015). Scaling prediction errors to reward variability benefits error-driven learning in humans. Journal of Neurophysiology, 114(3), 1628-1640.

[3]. Diederen, K. M., Spencer, T., Vestergaard, M. D., Fletcher, P. C., & Schultz, W. (2016). Adaptive prediction error coding in the human midbrain and striatum facilitates behavioral adaptation and learning efficiency. Neuron, 90(5), 1127-1138.

Reviewer #3

The main finding of this paper is that stimulus RPEs (quantified as mean value of presented options relative to either (a) mean of all options or (b) 0) drive an increase in risk seeking. The finding is nicely grounded in well-established models of basal ganglia function.

Short paper summary

Theory: After stimulus +RPE, people would ignore all below average rewards; this pulls the value of risky stimuli higher, away from their respective means

Main result: people are more risk seeking in the +RPE condition
Secondary result: people who have more RPE induced risk seeking (measured as degree to which PEIRS model wins over RW) show stronger pupillary response

We thank the reviewer for taking the time to read our paper so thoroughly, and for the very helpful comments.

Comments

1 The behavioural findings are interesting and clean, and the hypothesis clear

Thank you, we are glad that parts of our original manuscript were convincing. We hope that the revised version should be even clearer.

2 Could the effects be explained by attentionally-mediated higher learning rates in the high-risk condition? This would be similar to the RATES model (at least as I understood it), but with a dynamic rather than static individual-level learning rate; one possible way to test this: compute "empirical" learning rates from pupillary response, or "theoretical" learning rates from Eqs. 4 and 5

The reviewer suggests that an attention-based mechanism might cause the biases we observe. This is a very interesting idea, as similar effects have previously been mentioned in the context of risk preferences [1].

We followed the suggestion by devising a model in which learning is gated by surprise—the more surprising an outcome, the higher the effective learning rate. This was implemented by adding an additional surprise-related factor to the RW learning rule, the effect of which was controlled by a free parameter k:

$\Delta Q = \alpha \, |\delta|^k \, \delta$

For $k > 1$, more surprising outcomes will have more impact on the learner's estimate.

We found that this model, while interesting, could not explain the effects that we observed (see Fig 3D, Fig S5). We thus included it into our manuscript as a secondary alternative explanation, with a short mention in the main text, a dedicated methods section and additional analyses in the supplements. Overall, adding the attention-based model improved our manuscript by ruling out another possible alternative explanation.

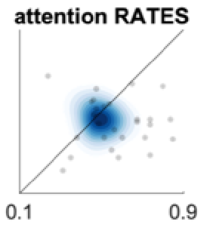

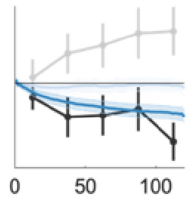

[1] Madan, C. R., Ludvig, E. A., & Spetch, M. L. (2014). Remembering the best and worst of times: Memories for extreme outcomes bias risky decisions. Psychonomic bulletin & review, 21(3), 629-636.

3 Please write out at least the complete winning models (PEIRS & PIRS) in their own separate box; as is, it's quite difficult to follow how different models build on one another

We quite liked the idea, but unfortunately there are no boxes in PLoS Comp B manuscripts. Instead, we created a separate section for PEIRS early in the manuscript. PIRS was deemphasized (see comments from reviewer 1), and does not feature prominently any more.

4 I didn't follow from the exposition what the authors expect the role of -RPEs to be for the both-low condition; and were trials of different conditions blocked? Would be good to state this upfront in the task figure and on lines 145-149

The both-low condition will provide a less than average reward of 50 (we have to choose between two bad stimuli, which will on average provide 40 points). When we learn that we are in the both-low condition, our reward expectation thus goes down, which causes a negative prediction error, which in turn makes us risk-averse.

The reviewer also correctly points out that we were not clear about how stimuli are selected in each trial. We added the missing information and modified the figure caption as well. In the results section we now say

> The stimuli shown on any given trial were selected pseudo-randomly, such that all ordered stimulus combinations (12 combinations) would occur equally often (10 times each) during each block.

5 Please be clearer early in the text that you mean stimulus prediction errors when you lay out the theory; on a first read I didn't catch until the discussion that the paper was attributing the risk effect to "stimulus prediction errors"

There is indeed a potential for confusion: both the outcome prediction error AND the stimulus prediction error are reward prediction errors. The outcome prediction error is difference between expected reward and received reward, the stimulus prediction error

is difference between reward expected before and after seeing the stimuli. We now clarify this immediately after introducing the stimulus prediction error by writing

> It is important to note that the stimulus prediction error is a reward prediction error that occurs at the time of stimulus onset, not an error in stimulus prediction. The identity of the stimulus is relevant only insofar as it is related to reward expectations.

and then again after introducing the outcome prediction error by writing

> Again, note that the outcome prediction error is a reward prediction error at the time of outcome presentation. In our parlance, stimulus prediction errors and outcome prediction errors exist within the same framework—they are both reward prediction errors but happen at different times.

In our manuscript, we thus only talk about changes in reward expectation, never about other forms of surprise, for example caused by a stimulus that rarely appears.

We further agree that our main proposal could be clearer early in the manuscript. The newly added section on the PEIRS model (suggested by this reviewer) should increase clarity substantially. Additionally, we are now also discussing a potential candidate model in which the previous outcome prediction error modulates risk-preferences. This should guide readers further towards the differences in what prediction errors are being used.

6 Re: discussion, it's hard to make inferences about dopamine from a behavioral task with eye-tracking. For instance, it's not clear whether the pupillary surprise signal is noradrenergically or dopaminergically modulated (Takeuchi … Morris 2016). Given the strong pupil effects authors observe, it might be that the surprise effect is driven by NE, which has classically been associated with the pupillary response, and with an unsigned "surprise" signal

The reviewer is very right in pointing out that our pupil analysis does not strengthen our claims we make about dopamine. After careful consideration, and also taking into

account the comments from the other two reviewers, we have thus decided to remove the pupil analysis from the main manuscript and only present it in the supplements. There, we also discuss the link between pupils, noradrenalin, surprise and prediction errors. The supplementary discussion reads

> We found pupil responses to the magnitude of both the stimulus prediction error and the outcome prediction error. Those might reflect the surprise associated with the two prediction errors that we have postulated and might hence constitute an indirect physiological correlate of the mechanisms we have described. Note that we do not claim that pupils should be thought of as a proxy for dopamine, in terms of a direct physiological link—in fact, pupils are perhaps more likely to reflect noradrenalin, see e.g. (Reimer, McGinley et al. 2016).
>
> Noradrenalin has been linked to uncertainty in an influential model (Yu and Dayan 2005). In that model, two types of uncertainty are identified: expected uncertainty (known unreliability of cues) and unexpected uncertainty (surprises following previously reliable cues). Expected uncertainty is associated with acetylcholine, which is discussed in the context of cortical and hippocampus learning. Unexpected uncertainty is linked to noradrenalin. The uncertainty in our task is of the expected variety—our theory even suggests that participants learn how reliable cues are with respect to the reward they predict. Unexpected uncertainty could be introduced into our task through reversals or other sudden changes but does not feature in the current design. We still detect pupil responses to (expectedly) surprising outcomes. Since pupils are linked to noradrenalin, it appears that our results are slightly at odds with the model of (Yu and Dayan 2005). Further work is required to dissect the different kinds of uncertainty, risk and surprise, the role of the various neurotransmitters and the relation to pupil dilation.
>
> For the intends and purposes of this study, we propose that pupil responses might reveal cognitive states such as surprise, which in turn are related to DA release

according to the widely accepted reward prediction error hypothesis. The link between pupil traces and DA is thus weak, which is why this analysis is a supplement to our main results, rather than a main result itself. Nevertheless, the pupil results together with our other findings yield a consistent picture.

7 Relatedly, could the effect be mediated by memory effects? Recent work has linked surprise to better memory, irrespective of the sign of RPEs (Rouhani … Niv 2018)

This is a fascinating idea. We believe that such effects would be well captured by the models variance RATES and attention RATES (the later suggested by the reviewer). Our model comparison and simulations seem to indicate that memory effects like the ones mentioned are distinct from the effects that drive risk preferences in our task.

We have added a paragraph in the discussion section, discussion memory theories as potential explanations. Our new discussion reads

Even though it not feasible to represent the memory buffer model in our framework, our inverse s-shaped UTIL model does capture the idea of overweighting of extreme experiences. The simulations of that model (Fig S5) show that such overweighting can indeed reproduce the risk preferences that we observed, which ties in well with the results of (Madan, Ludvig et al. 2014). However, our model selection procedure (Fig 3D) suggests that PEIRS still explains the data better. One big difference between the two explanations is that risk preferences can flexibly appear and disappear in PEIRS. The memory buffer theory (and equivalently the inverse s-shaped UTIL model) on the other hand attribute them to distortions in the learned values, and hence predict more persistent preferences.

Another potentially relevant phenomenon based on memory effects was reported recently: (Rouhani, Norman et al. 2018) show that both reward tracking itself, as well

as episodic memory, are enhanced in high-risk environments. In our context, this might mean that learning is boosted for stimuli that regularly produce large prediction errors, i.e. the risky stimuli, relative to the safe stimuli, either through directly boosting the reward learning process, or through boosted memory replay as described above. We included two models that could capture such effects: the variance RATES model allowed different learning rates for risky and safe stimuli, while in the attention RATES model, surprise could boost learning in all conditions. However, neither of the two models could explain the effects we observed, suggesting that the fascinating effects they describe are distinct from the effects that drive risk preferences in our experiment.

8 It would be helpful to place this work in the context of the Yu and Dayan expected vs. unexpected uncertainty model

We have added a paragraph on this model in the discussion of the pupil results, where we also discuss the reviewer's earlier point about noradrenalin (which is reproduced above).

Thanks for an interesting read!

You are very welcome! It is us who have to say thank you for many excellent suggestions, which lead to a strongly improved manuscript.
